# Effects of *Erbuzhuyu* Decoction Combined with Acupuncture on Endometrial Receptivity Are Associated with the Expression of miR-494-3p

**DOI:** 10.1155/2020/9739672

**Published:** 2020-11-25

**Authors:** Lan Yuan, Fen Feng, Zhu Mao, Jinzhu Huang, Yi Liu, Yulin Li, Rongxing Jiang

**Affiliations:** ^1^School of Medical and Life Sciences/Reproductive & Women-Children Hospital, Chengdu University of Traditional Chinese Medicine, Chengdu, Sichuan 610041, China; ^2^Hospital of Chengdu University of Traditional Chinese Medicine, Chengdu, Sichuan 610041, China; ^3^School of Nursing, Chengdu University of Traditional Chinese Medicine, Chengdu, Sichuan 611137, China

## Abstract

**Background/Aim:**

*Erbuzhuyu* decoction (EBZYD) is a traditional Chinese medicine (TCM) formula and has been used in infertility treatment. Meanwhile, acupuncture is also used to treat female infertility. However, it is unclear whether EBZYD combined with acupuncture has better therapeutic effect. The aim of this study was to explore the effect of EBZYD combined with acupuncture and investigate its mechanism in superovulation mice.

**Methods:**

The mice received the treatment of EBZYD, acupuncture, EBZYD combined with acupuncture, or miR-494-3p agomir combined with EBZYD and acupuncture. The blastocysts' number, endometrial microstructure, and endometrial thickness were observed, followed by the detection of endometrial receptivity-related factors, PI3K/Akt/mTOR pathway-related proteins, and miR-494-3p expression using quantitative real-time polymerase chain reaction (qRT-PCR) or western blot. Luciferase reporter assay was performed to confirm the targeting relationship between HOXA10 and miR-494-3p.

**Results:**

EBZYD combined with acupuncture treatment could increase the number of blastocysts, pinopodes, endometrial thickness, and the expression of endometrial receptivity-related factors, and the treatment effect of EBZYD combined with acupuncture was better than EBZYD or acupuncture alone. In addition, EBZYD combined with acupuncture treatment activated PI3K/Akt/mTOR pathway and inhibited the expression of miR-494-3p. HOXA10 is one of the target genes of miR-494-3p. Overexpression of miR-494-3p reversed the therapeutic effect of EBZYD combined with acupuncture and suppressed the expression of HOXA10 and the activity of PI3K/Akt/mTOR pathway.

**Conclusion:**

This study suggests that EBZYD combined with acupuncture could improve endometrial receptivity in superovulation mice via miR-494-3p/HOXA10 axis.

## 1. Introduction

In recent years, infertility patients have shown an increasing trend. Infertility has become a global medical and sociological issue affecting human development and health [1]. In recent years, in *vitro* fertilization-embryo transfer (IVF-ET) technology has developed rapidly, but its success rate is still low. Endometrial receptivity is an important factor that affects the success of IVF-ET [2]. Therefore, it is necessary to study the mechanisms of endometrial receptivity and improve implantation rate.

Several factors have been reported to be associated with endometrial receptivity. For instance, vascular endothelial growth factor (VEGF) is one of the key factors for angiogenesis and has been shown to improve endometrial receptivity by promoting angiogenesis [3]. HOXA10 is also an important factor affecting endometrial receptivity, which is essential for embryo adhesion [4, 5]. Previous study has found that mice with HOXA10 knocked out suffering endometrial receptivity damage and failed embryo implantation [6]. Besides, there is evidence that HOXA10 could activate PI3K/Akt pathway [7, 8]. PI3K/Akt/mTOR is a classic signaling pathway that has been shown to regulate cell proliferation and growth [9]. Previous studies have found that the PI3K/Akt/mTOR signaling pathway could regulate endometrial epithelial cell proliferation and capillary endometrial epithelial cell apoptosis to improve endometrial receptivity [10, 11].

MicroRNAs (miRNAs) are a class of short noncoding RNAs composed of 19 to 25 nucleotides in length and involve in posttranscriptional gene regulation [12]. In recent years, many miRNAs have been reported to regulate endometrial receptivity. miR-182 has been reported to downregulate PTN to participate in the development of endometrial receptivity [13]. A previous study has reported that overexpression of miR-29c reduced collagen type IV alpha 1 (COL4A1) mRNA expression to damage the adhesive capacity of the endometrium [14]. In addition, miR-494-3p has been reported to be downregulated in receptive endometria, but the mechanism is unclear [15].

*Erbuzhuyu* decoction (EBZYD) is a traditional Chinese medicine (TCM) formula widely used to treat infertility. EBZYD has been proved to improve endometrial receptivity [16, 17]. Acupuncture is also used to treat female infertility and has been shown to affect the menstrual cycle, improve endometrial morphology, and promote the microcirculation to heighten reproductive function [18–22]. A systematic review and meta-analysis has shown that acupuncture could improve the pregnancy rate and embryo transfer rate and thickening endometrium, but the effect based on the use of acupuncture is weak [23]. In this study, we hypothesized that EBZYD combined with acupuncture has better therapeutic effect and explored its potential mechanism.

## 2. Materials and Methods

### 2.1. Composition of EBZYD

EBZYD is made up of Rhizoma Drynariae, Fructus Psoraleae, *Morinda officinalis*, Mulberry parasite, *Dipsacus asperoides*, medicinal *Cyathula* root, Caulis Spatholobi, and Radix Curcumae. The granule preparation was purchased from Beijing University of Chinese Medicine.

### 2.2. Animal Model Generation and Treatment

Refer to the relevant literature to establish a model of superovulation [24]. 8-week-old C57BL6 mice (female = 90, male = 25) were provided by Chengdu Dashuo Experimental Animal Co., Ltd. In the first stage, mice were randomly divided into the blank control group, the model group, the EBZYD group, the acupuncture group, and the EBZYD combined with acupuncture group after 2 estrous cycles, with 10 female mice in each group. The model group, the EBZYD group, the acupuncture group, and the EBZYD combined with acupuncture group were injected intraperitoneally with gonadotropin releasing hormone agonists (GnRHa) 40 *μ*g/100 g for 9 d. On the 9th day, human menopausal gonadotropin (HMG) 40 IU/100 g was injected, and human chorionic gonadotropin (HCG) 100 IU/100 g was injected 48 hours later. After 5 d of continuous injection of GnRHa, the vaginal exfoliated cell smear showed no obvious changes in the cycle, showing the performance of the estrous interval, and a large number of white blood cells were seen under the microscope. After the injection of HMG and HCG, vaginal secretions increased significantly, and the smear of vaginal exfoliated cells showed a large number of nonnucleated keratinocytes, which means that the model was successful. The EBZYD group and the EBZYD combined acupuncture group were given EBZYD at a dose of 30 g/kg/d for 11 days when modeling started. In the acupuncture group and the EBZYD combined acupuncture group, acupuncture treatment was performed once a day for 11 days at acupuncture points *Guanyuan* (BL26), *Sanyinjiao* (SP6), and *Senshu* (BL23). The needles were inserted to depth of 3 mm and twirled every 3 min; the needles were retained for 10 min. In the second stage, mice were randomly divided into the blank control group, the model group, the EBZYD combined with acupuncture group, the miR-494-3p agomir combined with EBZYD, and acupuncture group after 2 estrous cycles, with 10 female mice in each group. The modeling method is the same as the first stage. For treatment strategy, the miR-494-3p agomir combined with EBZYD and acupuncture group were treated with 10 mg/kg miR-494-3p agomir (miR40003182-4-5, RiboBio Co., Ltd., China) for once by intraperitoneal injection; beginning 15 min after establishment of the model, the subsequent treatment is the same as the EBZYD combined with acupuncture group. Then, put the female and male mice together at the ratio of 2 : 1 and observe the vaginal smear next morning. Those who found sperm were recorded as pregnancy D1. At pregnancy D5, mice were anesthetized with 3% pentobarbital sodium injection, and the uterus of the mice was immediately removed.

### 2.3. Pathological Examination

The endometrium tissues were fixed with 4% paraformaldehyde for 48 h and prepared for 5-*μ*m-thick paraffin sections. Then, endometrium tissues were stained using the HE staining kit (Beyotime, China) and observed under the AE31 microscope (MOTIC, Canada). Ultrastructural changes of endometrium were observed using the BA600Mot scanning electron microscope (SEM) (MOTIC, Canada). Briefly, the endometrium tissues were prefixed with 2.5% glutaraldehyde. Following this, they were dehydrated with acetone and soaked in isoamyl acetate for 2 h. Then, vacuum drying and metal coating were used for the tissue treatment. The treated tissues were observed by SEM.

### 2.4. Quantitative Real-Time PCR (qRT-PCR)

Total RNA of uterus was isolated using TRIzol reagent (Invitrogen, USA). Mir-X miRNA qRT-PCR TB Green® Kit and the TB Green® Fast qPCR Mix (TaKaRa, Japan) were performed to detect the expression of miR-494-3p and HOXA10 with the following conditions: 3 min of predenaturation at 95°C, then 5 s of denaturation at 95°C and 30 s of annealing/extension at 60°C for 40 cycles. The qRT-PCR results were analyzed as the fold change (2^−ΔΔCt^). U6 and GAPDH were used for the normalization of miRNA and mRNA, respectively. The primer sequences are shown in Table 1.

### 2.5. Western Blot

The total protein was extracted from the uterus by using RIPA lysis buffer (Beyotime, China) and quantified with bicinchoninic acid (BCA) protein assay kit (Thermo Fisher, USA). An equal amount of protein was separated by 15% SDS-PAGE and transferred onto PVDF membranes (Millipore, USA). The PVDF membranes were blocked with 5% skimmed milk for 1 h and then incubated with primary antibodies against HOXA10 (ab191470, 1:1000, Abcam, UK), Akt (ab8805, 1:1000, Abcam), p-Akt (ab38449, 1:1000, Abcam), mTOR (ab2732, 1:2000, Abcam), and p-mTOR (ab109268, 1:1000, Abcam) at 4°C overnight. After that, the membranes were incubated with horseradish peroxidase- (HRP-) labeled secondary antibody (ab205718, 1:2000, Abcam, UK) at room temperature for 1 hour. Protein bands were visualized with efficient chemiluminescence (ECL) kits (Solarbio, China).

### 2.6. Cell Culture and Dual-Luciferase Reporter Assay

Wild-type (Wt) or mutant (Mut) fragments of the 3′-untranslated region (3′-UTR) from HOXA10 which including the potential miR-494-3p binding site were synthesized by Hanbio Co., Ltd. (China). Then, the synthesized fragments were connected into the psiCheck2 Vector (Promega, USA). For luciferase reporter assay, HEK-293T cells (Procell Life Science & Technology Co., Ltd., China) were seeded at 1 × 10^4^ cells/well in 96-well plates and cultured in Dulbecco's modification of Eagle's medium Dulbecco (DMEM) (Gibco, USA) containing 10% fetal calf serum (FBS) (Gibco) and 1% Penicillin-Streptomycin (Gibco) and placed in a humidified 37°C, 5% CO_2_ cell incubator for 24 h. Then, cells were cotransfected with csbml'5 pmol of miR-494-3p mimic/NC mimic and 0.16 *μ*g of HOXA10-Wt/Mut recombinant plasmids. The Dual-Luciferase Reporter Assay System (Promega, USA) was used to monitor the luciferase activity after transfection for 48 h.

### 2.7. Statistical Analysis

Data were presented as means ± standard deviation (SD). Statistical evaluations and calculations were evaluated by SPSS 22.0 (IBM, USA). Differences of data were tested by using Student's *t*-test or one-way analysis of variance (ANOVA) with the least significant difference (LSD) test, and *P* < 0.05 was considered a statistically significant difference.

## 3. Results

### 3.1. EBZYD Combined with Acupuncture Improves Endometrial Receptivity

**Figure 1:**
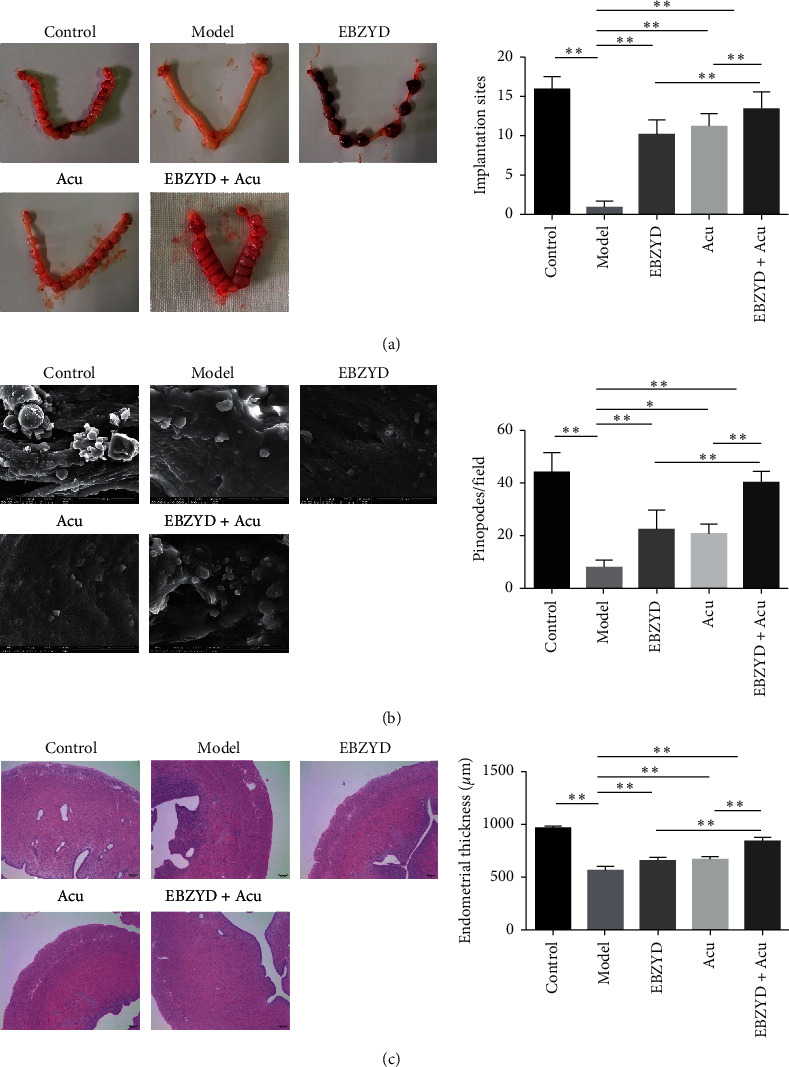
The effect of EBZYD combined with acupuncture treatment on endometrial receptivity. Control: the control group; Model: the model group; EBZYD: the EBZYD treatment group; Acu: the acupuncture treatment group; and EBZYD + Acu: the EBZYD combined with acupuncture treatment group. (a) The comparison of blastocyst number among each group; (b) endometrial surface detected by SEM; and (c) the comparison of endometrial thickness among each group. ^*∗*^*P* < 0.05; ^*∗∗*^*P* < 0.01.

As shown in Figure 1(a), significant differences in blastocyst number can be found between model and control groups. Compared with the model group, the number of blastocysts in the EBZYD, acupuncture, and EBZYD combined with acupuncture groups were significantly increased.  Figure 1(b) shows a scanning electron microscope (SEM) image of the endometrial surface. The number of blastocysts in the EBZYD combined with acupuncture group was markedly higher than that in the EBZYD group and the acupuncture group. Meanwhile, there were a major number of pinopodes on the endometrial surface in the control and EBZYD combined with acupuncture groups. Pinopodes existed in a part of endometrium in the EBZYD and acupuncture groups, while few pinopodes can be found in the model group. The HE staining results showed in Figure 1(c), compared with the control group, that the endometrial thickness in the model group was significantly decreased. The endometrial thickness significantly increased in the EBZYD, acupuncture, and EBZYD combined with acupuncture groups compared with the model group. Moreover, compared with the EBZYD and acupuncture groups, the endometrial thickness in the EBZYD combined with acupuncture group was markedly increased. These results indicated that EBZYD combined with acupuncture could improve endometrial receptivity.

### 3.2. Endometrial Receptivity-Related Factors Expression Is Increased upon EBZYD Combined with Acupuncture Treatment

**Figure 2:**
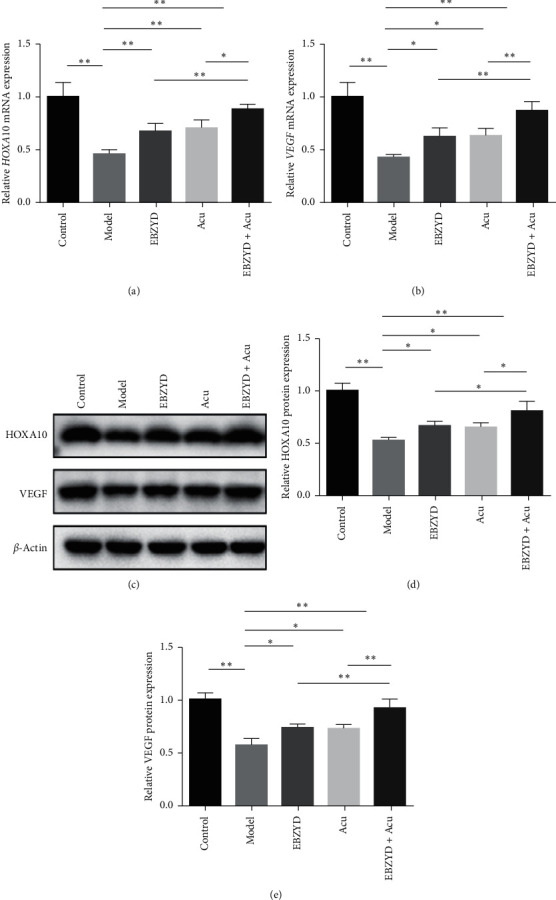
The effect of EBZYD combined with acupuncture treatment on the expression of endometrial receptivity-related factors. Control: the control group; Model: the model group; EBZYD: the EBZYD treatment group; Acu: the acupuncture treatment group; and EBZYD + Acu: the EBZYD combined with acupuncture treatment group. (a) The relative expression of *HOXA10* detected by qRT-PCR; (b) the relative expression of *VEGF* detected by qRT-PCR; (c) HOXA10 and VEGF protein bands image; (d) the relative expression of HOXA10 detected by WB; and (e) the relative expression of VEGF detected by WB. ^*∗*^*P* < 0.05; ^*∗∗*^*P* < 0.01.

The result of quantitative RT-PCR was shown in Figures 2(a) and 2(b). Compared with the control group, the expression levels of *HOXA10* and *VEGF* in the model group were significantly decreased. Compared with the model group, the expression levels of *HOXA10* and *VEGF* were significantly increased in the EBZYD, acupuncture, and EBZYD combined with acupuncture groups, while the expression levels of *HOXA10* and *VEGF* significantly increased in the EBZYD combined with acupuncture group compared with the EBZYD and acupuncture groups. In addition, we obtained the same results in western blot experiments (Figures 2(c)–2(e)). These results demonstrated that EBZYD combined with acupuncture treatment could improve endometrial receptivity by increasing the expression of HOXA10 and VEGF.

### 3.3. PI3K/Akt/mTOR Pathway Is Activated by EBZYD Combined with Acupuncture Treatment

**Figure 3:**
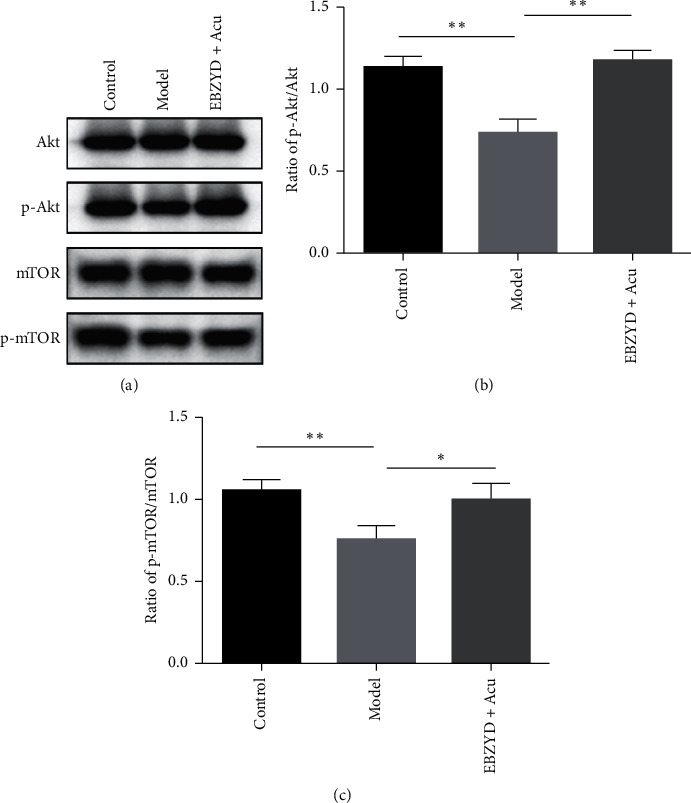
The effect of EBZYD combined with acupuncture treatment on the expression of PI3K/Akt/mTOR pathway related proteins. Control: the control group; Model: the model group; and EBZYD + Acu: the EBZYD combined with acupuncture treatment group. (a) Akt, p-Akt, mTOR, and p-mTOR protein bands image; (b) the ratio of p-Akt/Akt; and (c) the ratio of p-mTOR/mTOR. ^*∗*^*P* < 0.05, ^*∗∗*^*P* < 0.01.

Compared with the control group, the levels of p-Akt/Akt and p-mTOR/mTOR in the model group were significantly decreased, while compared with the model group, the ratios of p-Akt/Akt and p-mTOR/mTOR were significantly increased in the EBZYD combined (Figures 3(a)–3(c)). These results demonstrated that EBZYD combined with acupuncture treatment might improve endometrial receptivity by activating the PI3K/Akt/mTOR signaling pathway.

### 3.4. EBZYD Combined with Acupuncture Inhibits the Expression of miR-494-3p Which Targets HOXA10

**Figure 4:**
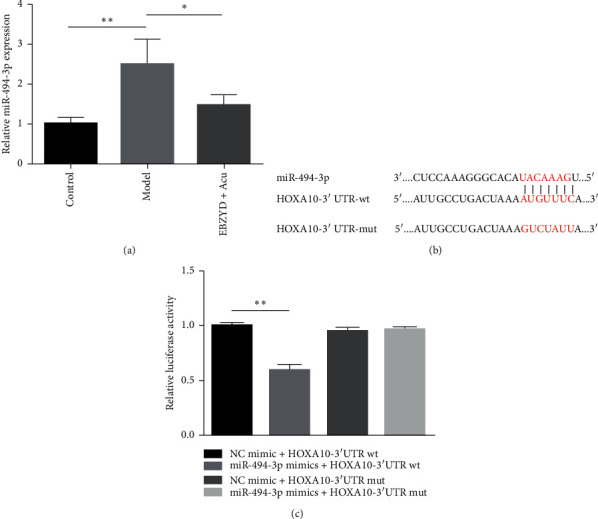
The effect of EBZYD combined with acupuncture treatment on the expression of miR-494-3p, which has a potential targeting relationship with HOXA10. Control: the control group; Model: the model group; and EBZYD + Acu: the EBZYD combined with acupuncture treatment group. (a) The relative expression of miR-494-3p detected by qRT-PCR; (b) the sequence of binding sites between HOXA10-3′UTR and miR-494-3p; and (c) HOXA10-3′UTR wt or HOXA10-3′UTR mut connected to the luciferase coding region and transfected in 293T cells with miR-494-3p mimic or NC mimic to confirm HOXA10 is the target of miR-494-3p. ^*∗*^*P* < 0.05, ^*∗∗*^*P* < 0.01.

To further explore the molecular mechanism of EBZYD combined with acupuncture treatment, the expression of miR-494-3p was detected by qRT-PCR. As shown in Figure 4(a), compared with the control group, the expression of miR-494-3p was significantly reduced in the model group, while the expression miR-494-3p was significantly increased in the EBZYD combined with acupuncture group compared with the model group. To investigate the biological mechanism of miR-494-3p in endometrial receptivity, TargetScan was performed to predict the potential targets of miR-494-3p. The results showed that the 3ʹUTR of HOXA10 contains a putative miR-494-3p binding site (Figure 4(b)). To confirm the potential targeting relationship between miR-494-3p and HOXA10, a Dual-Luciferase Reporter Assay was performed. miR-494-3p mimic or Negative Control (NC) mimic and psiCheck2-HOXA10-3′UTR wt or mut were cotransfected into 293T cells. The data showed that miR-494-3p mimic decreased luciferase activity of HOXA10-3′UTR wt, rather than HOXA10-3′UTR mut (Figure 4(c)). These results indicated that HOXA10 is the target gene of miR-494-3p.

### 3.5. Overexpression of miR-494-3p Reversed the Treatment Effect of EBZYD Combined with Acupuncture

**Figure 5:**
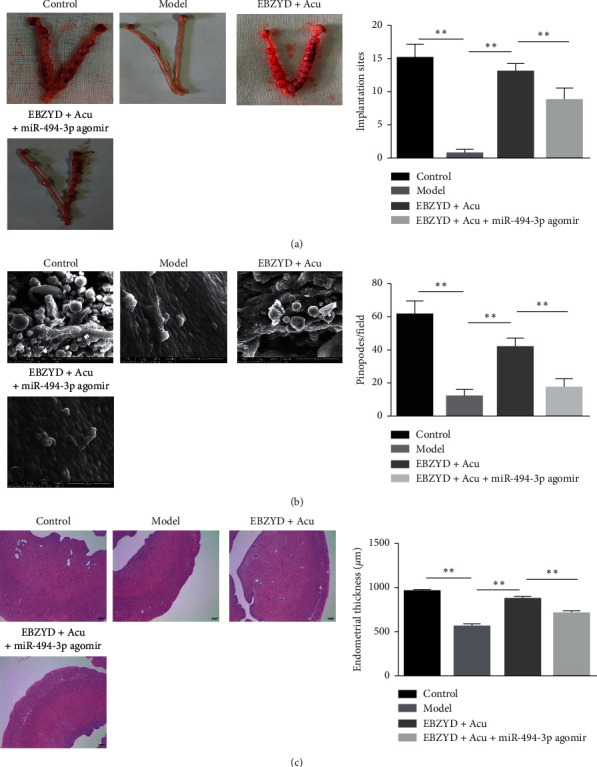
The effect of miR-494-3p on the ameliorative endometrial receptivity treated by EBZYD combined with acupuncture. Control: the control group; Model: the model group; EBZYD + Acu: the EBZYD combined with acupuncture treatment group; and miR-494-3p agomir + EBZYD + Acu: miR-494-3p agomir combined with EBZYD and acupuncture treatment group. (a) The comparison of blastocyst number among each group; (b) endometrial surface detected by SEM; and (c) the comparison of endometrial thickness among each group. ^*∗*^*P* < 0.05, ^*∗∗*^*P* < 0.01.

As shown in Figure 5(a), compared with the EBZYD combined with acupuncture group, the number of blastocysts in the miR-494-3p agomir administration group was significantly decreased. Meanwhile, the number of pinopodes on the endometrial surface in the miR-494-3p agomir administration group was less than that in the EBZYD combined with acupuncture group (Figure 5(b)). The HE staining results showed in Figure 5(c), compared with the EBZYD combined with acupuncture group, that the endometrial thickness in the miR-494-3p agomir administration group was significantly decreased. These data demonstrated that overexpression of miR-494-3p could reverse the ameliorative endometrial receptivity treated by EBZYD combined with acupuncture.

### 3.6. Overexpression of miR-494-3p Inhibited the Enhanced HOXA10 Expression and the Activated PI3K/Akt/mTOR Pathway by EBZYD Combined with Acupuncture Treatment

**Figure 6:**
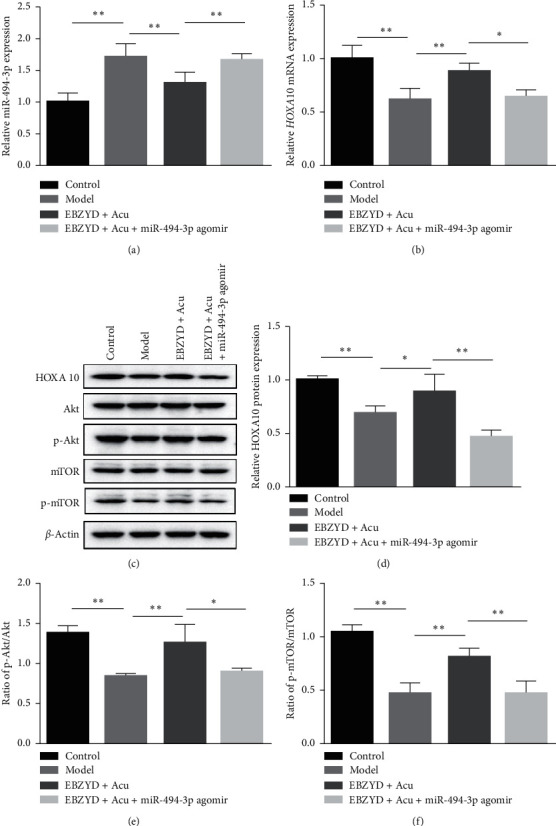
The effect of miR-494-3p on the expression of HOXA10 and PI3K/Akt/mTOR pathway-related proteins. (a) The relative expression of miR-494-3p detected by qRT-PCR; (b) the relative expression of *HOXA10* mRNA detected by qRT-PCR; (c) HOXA10, Akt, p-Akt, and mTOR; (d) p-mTOR protein bands image; (e) the ratio of p-Akt/Akt; and (f) the ratio of p-mTOR/mTOR. ^*∗*^*P* < 0.05, ^*∗∗*^*P* < 0.01.

As shown in Figures 6(a)–6(d), compared with the EBZYD combined with acupuncture group, miR-494-3p agomir treatment increased the expression of miR-494-3p, while the expression of HOXA10 mRNA and protein significantly decreased in the miR-494-3p agomir administration group compared with the EBZYD combined with acupuncture group. Moreover, compared with the EBZYD combined with acupuncture group, the ratios of p-Akt/Akt and p-mTOR/mTOR in the miR-494-3p agomir administration group were observably decreased (Figures 6(e) and 6(f)). These data reminded that overexpression of miR-494-3p could reverse the enhanced expression of HOXA10 and the activated PI3K/Akt/mTOR pathway induced by EBZYD combined with acupuncture.

## 4. Discussion

As two methods TCM treatment of infertility, EBZYD and acupuncture have been proven to have a good curative effect in clinic, respectively [25–27]. The present study aimed to investigate the effect of EBZYD combined with acupuncture treatment on endometrial receptivity in superovulation mice. Our results indicated that the treatment effect of EBZYD combined with acupuncture was better than EBZYD or acupuncture alone. Previous studies have shown that acupuncture can improve the efficacy of Chinese herbal medicine in a variety of diseases [28–30]. We hypothesized that acupuncture and EBZYD also have synergistic effects in superovulation mice. In addition, EBZYD combined with acupuncture treatment could increase the number of blastocysts, pinopodes, and endometrial thickness in superovulation mice via miR-494-3p/HOXA10 axis and PI3K/Akt/mTOR pathway.

We observed the number of blastocysts and found that the superovulation mice had little ability of embryo implantation. EBZYD or acupuncture administration could improve the ability of embryo implantation, while EBZYD combined with acupuncture treatment could further improve the ability of embryo implantation. Concerning SEM observation and H&E staining, we found that the number of pinopodes and endometrial thickness were increased by EBZYD or acupuncture administration in superovulation mice. EBZYD combined with acupuncture treatment showed more pinopodes and thicker endometrium than EBZYD or acupuncture administration. The development and maturation of the vascular network are essential for the successful development of the placenta and normal embryonic growth [31]. VEGF is a key angiogenic modulator, regulating uterine angiogenesis [32, 33]. A previous study has shown that in mice with defective expression of VEGF would miscarry due to impaired uterine angiogenesis [34]. Xin et al. found that *Wenshen Yangxue* decoction could help endometrial receptivity recovery in rats and increased the expression of VEGF [35]. HOXA10 is a homeobox-containing transcription factor and has a high expression level during the window of implantation [36]. Wang et al. reported that 5-aza-2′-deoxycytidine could improve endometrial receptivity by increasing the expression of HOXA10 [37]. It is suggested that VEGF and HOXA10 play a crucial role in endometrial receptivity. In this study, we found that EBZYD, acupuncture, or EBZYD combined with acupuncture administration could upregulate the expression of VEGF and HOXA10. These results demonstrated that EBZYD combined with acupuncture treatment could improve endometrial receptivity and had a better treatment effect than EBZYD or acupuncture administration.

Then, we found that EBZYD combined with acupuncture treatment could activate PI3K/Akt/mTOR pathway. PI3K/Akt/mTOR pathway is an important signal cascade regulator of cell proliferation and has been shown to regulate the proliferation of endometrial epithelial cells [10]. *In vivo*, Kanchan et al. reported that Sorcin improves embryo implantation via PI3K/Akt signaling pathway in mice [38]. Moreover, there is evidence that HOXA10 could activate PI3K/Akt pathway in human hepatocellular carcinoma and chronic myelogenous leukemia [7, 8]. Here, we considered that EBZYD combined with acupuncture treatment might improve endometrial receptivity by upregulating the expression of HOXA10 to activate the PI3K/Akt/mTOR pathway.

Additionally, we found that miR-494-3p was overexpressed in the uterus of superovulation mice and reduced by EBZYD combined with acupuncture administration. miR-494-3p has been reported to be an oncogene that promotes tumor development [39, 40]. A past study conducted a microarray analysis of miRNAs related to endometrial receptivity and found that miR-494-3p was downregulated in receptive endometrium [15]. Our results suggest that the improvement of EBZYD combined with acupuncture administration in endometrial receptivity might be related to the downregulation of miR-494-3p. Further research found that HOXA10 is the target gene of miR-494-3p. Interestingly, overexpression of miR-494-3p *in vivo* could inhibit the increased HOXA10 expression and activated PI3K/Akt/mTOR pathway induced by EBZYD combined with acupuncture administration and reverse the therapeutic effect of EBZYD combined with acupuncture administration. It is suggested that the mechanism of EBZYD combined with acupuncture to improve endometrial receptivity might be to downregulate miR-494-3p, thereby increasing the expression of HOXA10 and activating the PI3K/Akt/mTOR pathway.

## 5. Conclusion

Our results demonstrated that EBZYD combined with acupuncture is more effective in improving endometrial receptivity than EBZYD or acupuncture treatment alone. EBZYD combined with acupuncture treatment could improve endometrial receptivity via miR-494-3p/HOXA10 axis and PI3K/Akt/mTOR pathway. Our research provides a novel treatment strategy for the failure of IVF-ET.

## Figures and Tables

**Table 1:** The primer information.

Gene	Sequences
miR-494-3p	Forward: ATCCAGTGCGTGTCGTG
	Reverse: Mir-X miRNA qRT-PCR TB Green^®^ kit provided
HOXA10	Forward: CTCTCTCCCCCTCACACTC
	Reverse: ACAAAACCACCAAAGCAAACACACA
U6	Mir-X miRNA qRT-PCR TB Green^®^ kit provided
GADPH	Forward: GGTTGTCTCCTGCGACTTCA
	Reverse: GGTGGTCCAGGGTTTCTTACT

## Data Availability

The datasets used or analyzed during the current study are available from the corresponding author upon reasonable request.
